# Videofluoroscopic evaluation of mastication and swallowing in individuals with TMD

**DOI:** 10.1590/S1808-86942012000400006

**Published:** 2015-10-20

**Authors:** Carla Maffei, Marçal Motta de Mello, Noemi Grigoletto de Biase, Lilian Pasetti, Paulo A. Monteiro Camargo, Kelly Cristina Alves Silvério, Maria Inês Rebelo Gonçalves

**Affiliations:** 1 PhD in Dentistry/Stomatology - PUC/PR; Speech and Hearing therapist - Digestive Motility Ward - Red Cross Hospital and São Vicente Hospital. Professor/collaborator - ENT Residency Program - Curitiba/PR); 2 Cardiologist. Expert in Oral Motility/Dysphagia - PUC/PR (General Practice - Physician in charge of the Digestive Motility Ward - Red Cross Hospital and São Vicente Hospital - Curitiba/PR); 3 PhD and Senior Associate Professor - Paulista School of Medicine - Federal University of São Paulo (UNIFESP/EPM); MD, ENT, Associate Professor - Speech and Hearing Therapy School - Pontifical Catholic University of São Paulo); 4 PhD in Medicine and Maxillo-Facial Surgery - Universidad Complutense de Madrid/Spain. Dentist, Head of the Maxillo-Facial Traumatology and Surgery of the Ecoville Hospital and Santa Cruz Hospital. Curitiba/PR); 5 PhD in Clinics and Surgery - UFPr. ENT physician. Head of the Larynx Department and the Medical Residency Program at the Angelina Caron Hospital. Director of the ENT Ward and Neck-and-Facial Surgery Ward of the Centro Avançado de ORL - Curitiba/PR); 6 PhD in Sciences - Dental School of Piracicaba - FOP/UNICAMP; Speech and Hearing Therapist, Expert in Voice and Orofacial Motility. Professor at the Department of Speech and Hearing Therapy of the Dental School of Bauru - University of São Paulo - FOB/USP); 7 Post-Doctoral degree from the University of California, Davis/USA; Speech and Hearing Therapist; Professor at the Department of Speech and Hearing Therapy - UNIFESP/EPM; 8 Head of the Integrated Speech and Hearing Therapy Ward of the São Paulo Hospital. Head of the Speech and Hearing Therapy Program at the IOP/GRAACC/UNIFESP). Universidade Tuiuti do Paraná

**Keywords:** deglutition disorders, temporomandibular joint, temporomandibular joint dysfunction syndrome

## Abstract

To study mastication and swallowing disorders in patients with temporomandibular disorders (TMD).

**Objective**: To investigate mastication and swallowing disorders in patients with severe TMD referred to surgery.

**Materials and Methods**: Clinical and experimental study involving ten individuals with TMD submitted to deglutition videofluoroscopy. These patients did not have posterior teeth, mastication pain and food replacement in favor of pasty consistence food. The assessment of the oral and pharyngeal phases approached the following aspects: side of onset and preferential side for chewing, premature escape, remains of food residues in the oral cavity or in the pharyngeal recesses, number of necessary swallowing efforts, laryngeal penetration and/or tracheal aspiration.

**Results**: During mastication and the oral phase we observed tongue compensatory movements upon chewing (n = 7; 70%), premature escape (n = 4; 40%), food remains in the cavity after swallowing (n = 5; 50%) and an excessive number of deglutition efforts (n = 5; 50%). On the pharyngeal phase we observed food remains in the valleculae (n = 6; 60%), in the pyriform sinuses (n = 4; 40%); laryngeal penetration (n = 1; 10%) and tracheal aspiration (n = 4; 40%).

**Conclusion**: TMD patients may have alterations in their chewing and swallowing patterns, with laryngeal penetration and/or tracheal aspiration. The study indicates the need for a multidisciplinary assessment because of dysphagia in TMD patients.

## INTRODUCTION

Temporomandibular dysfunction (TMD) is a generic term which describes a broad array of changes to the masticatory and temporomandibular joint (TMJ) muscles. It is a multifactorial condition which may be acquired by one or more factors, such as occlusal unbalance, stress and rheumatic or neuromuscular systemic diseases, which may manifest alone or in different associations^1^, ^2^, ^3^.

Most TMD-related complaints include pain, especially in the masticatory muscles, pre-auricular region and/or the very TMJ region. Pain may alter normal muscle function due to an increase in the afferent excitatory stimuli. Affected individuals may have limitations or asymmetries in the execution of mandibular movements, crackling sounds during mandible opening and closure, including during speech. The prevalence of TMD signs and symptoms in the general population varies between 40% and 60%, which justifies the scientific interest on this disorder, especially concerning the professionals dedicated to the propaedeutic as well as the prevention and clinical aspects associated with the different types of treatment and rehabilitation for the stomatognathic system^4^, ^5^.

The precise TMD etiology is, often times, a major challenge, since the manifestation of similar symptoms may be associated with different anatomical and functional alterations^5^. Masticatory and swallowing are, in general, the most altered functions in these patients.

Videofluoroscopy is the gold standard test utilized to assess swallowing, being paramount for the diagnosis of dysphagia, showing the possible risks involved in per os feeding, which cause repetitive bronchoaspiration pneumonia. Such technique also enables the finding of compensatory movements during chewing, arising from the TMD^6^.

The scientific literature has reports on alterations in tongue position and the presence of compensatory neck movements to aid mastication in TMD patients^7^, calling the attention of healthcare professionals concerning the presence of choking during mastication and deglutition^8^, ^9^. Insofar as the assessment of mastication goes, studies have recorded the presence of unbalance in the chewing muscles, in which there is only one side working (chewing), requiring changes in food consistence, and pain during the execution of such task^10^, ^11^. Despite these clinical observations, none of these studies utilized videofluoroscopy as means to collect further information and details on the functions of mastication, as well as deglutition phases.

Masticatory and deglutition assessment in TMD patients using fluoroscopy enables a broad spectrum analysis for the healthcare professional. It is extremely useful in the dynamic assessment of the physiological movements involved in such tasks, as well as oropharyngeal compensatory motor adjustments. It also guides in the setting up of exercise programs for the stomatognathic system, postural and finger maneuvers with the goal of correcting changes in mastication and deglutition^12^.

Thus, the goal of the present paper was to investigate and find masticatory and/or swallowing alterations in individuals with TMD with indications for surgical correction, by means of videofluoroscopy.

## MATERIALS AND METHODS

There were ten individuals participating in this study: six women and four men, aged between 20 and 34 years, diagnosed with TMD and in a pre-surgical treatment stage. They all signed an informed consent form and the study was approved by the Ethics in Research Committee of the aforementioned institution, under protocol # 37/2003.

The patients' main complaints included: reduction of mouth opening range of motion and referred pain on the chewing and neck-facial muscles. In six individuals there were one or two molar teeth missing in one or two sides, and there was also the need to change the food, with a food preference for liquid and pasty types of food, refusing solid types.

The tasks' videofluoroscopy exams were carried out using a RADIUS image intensifier, a pro-cision 5 Head System compact JVC VHS video-camera with a color liquid-crystal screen, 50x digital zoom and Triton tripod. The tests were recorded and digitalized. Later on, the images were analyzed in a frame-by-frame basis. The exams were carried out by a physician and two speech therapists, all specialized in deglutition disorders. The patients were kept seating down at an angle of approximately 90°, keeping regular neck posture and with their feet fully supported on the ground - Frankfurt plane. The equipment was geared towards capturing images of the lower third of the face and neck, and the images were recorded in side and anteroposterior views^13^.

Liquid (1 ml, 5 ml and 20 ml), pasty (5 ml) and solid food were given, in this order. The liquid consistency was obtained by diluting 20 ml of water and 20 ml of barium sulphate (Bariogel®); the pasty consistency was obtained by mixing 20 ml of barium sulphate, 20 ml of water and 9 g (two soap spoons) of liquid thickener (Nutylis, Support®). The solid food (bread) was given in pieces of approximately 1 cm^2^, also dyed with barium sulphate. The patients were instructed to chew and swallow the food according to their habitual pattern.

The oral and pharyngeal phases of swallowing were analyzed for all the consistencies. During the oral phase we investigated: the presence of premature oral food escape, food residues in the oral cavity, besides the number of necessary swallowing movements for the complete emptying of the oral cavity - more than three sequential swallowing attempts were considered as an alteration. In the pharyngeal phase we investigated laryngeal and hyoid bone anteriorization and elevation movements during swallowing, food residue in the valleculae and pyriform sinuses, laryngeal penetration and tracheal aspiration.

As far as mastication is concerned, we assessed the presence of compensatory tongue movements, defined as a holding characteristic of the food bolus against the hard palate and anterior incisive teeth, in an attempt to promote a better grinding of the food bolus. In analyzing the images in the anteroposterior position the initial side of mastication and the preferred side were identified (right, left or anterior). The results were statistically treated by means of simple percentages.

## RESULTS

As to the preferred chewing side, 90% of the individuals had the right side as preferred (Chart 1), with the concurrent presence of compensatory tongue movements.

**Chart 1:** Videofluoroscopic findings in TMD patients (n = 10) during the oral phase of deglutition: without alteration, premature food escape, persistence of residues in the oral cavity, excessive number of swallowings for the different food consistencies and volumes.

I	WTC	PE	ROC	END/CV
1	Liquid: 1 ml, 5 ml, 20 ml; Pasty; Solid			4/Liquid: 20 ml
2	Liquid: 1 ml, 5 ml; Pasty; Solid	Liquid: 20 ml		4/Liquid: 20 ml; Solid
3	Liquid: 5 ml	Liquid: 1 ml, 20 ml	Pasty; Solid	
4	Liquid: 1 ml, 5 ml		Liquid: 20 ml; Pasty; Solid	4/Solid
5	Liquid: 1 ml, 5ml, 20 ml; Pasty		Solid	4/Liquid: 20 ml; Pasty; 8/Solid
6	Liquid: 1 ml, 5 ml, 20 ml; Pasty; Solid			
7	Liquid: 1 ml, 5 ml, 20 ml; Pasty; Solid			
8	Liquid: 1 ml, 5 ml, 20 ml; Pasty; Solid			
9		Liquid: 1 ml, 5 ml, 20 ml; Pasty; Solid	Solid	
10		Liquid: 1 ml, 5 ml, 20 ml; Pasty; Solid	Liquid: 1 ml, 5 ml, 20 ml; Pasty; Solid	4/Liquid 20 ml; 5/Solid

I: Individuals; WTC: Without changes; PE: premature escape; ROC: Residue in the oral cavity; END/CV: Excessive number of deglutition efforts; ml: milliliter.

During swallowing assessment, 70% of the patients had three types of alterations (Charts 1 and 2).

**Chart 2:** Videofluoroscopy exam findings in TMD patients (n = 10) during the pharyngeal phase of deglutition: without changes, presence of residue in the valleculae, pyriform sinuses, laryngeal penetration and tracheal aspiration, considering each consistency and food volume.

I	WtC	RV	RPS	LP	TA
1	Liquid: 1 ml, 5 ml, 20 ml	Pasty, Solid			
2	Pasty, Solid			Liquid: 1 ml, 5 ml, 20 ml	Liquid: 20 ml
3	Liquid: 1 ml; Solid	Liquid: 5 ml, 20 ml; Pasty	Liquid: 20 ml		Liquid: 20 ml
4	Liquid: 1 ml	Liquid: 5 ml, Pasty, Solid	Liquid: 5 ml, Pasty		
5	Solid	Liquid: 1 ml, 5 ml, 20 ml; Pasty	Liquid: 20 ml		Liquid: 20 ml
6	Liquid: 1 ml, 5 ml, 20 ml; Pasty, Solid				
7	Liquid: 1 ml, 5 ml, 20 ml; Pasty, Solid				
8	Liquid: 1 ml, 5 ml, 20 ml; Pasty, Solid				
9	Liquid: 1 ml; Solid	Liquid 5 ml, 20 ml; Pasty			
10		Liquid 1 ml, 5 ml, 20 ml; Pasty, Solid	Liquid: 1 ml, 5 ml, 20 ml; Pasty		Liquid: 20 ml

I: Individual; WTC: Without changes; RV: Residue in the valleculae; PS: Residue in pyriform sinuses; LP: Laryngeal penetration; TA: Tracheal aspiration; ml: milliliter.

Premature food escape, established by the presence of contrasted food in the region of the pharyngeal recesses before triggering swallowing reflex, was observed during the swallowing of liquid and solid types of food in 40% of the individuals (Chart 1).

We found the persistence of food residue in the oral cavity of 50% of the cases, 10% for liquids in the volume of 20 ml; pasty and solid consistencies, 10%; 20% for solid; 10% for all the consistencies and volumes utilized (Chart 1).

## DISCUSSION

The results from the present study may be associated with the missing of posterior teeth and/or the use of misfit dental prosthesis, the reduction in intraoral proprioception mechanisms, the pain present in the orofacial muscles which lead the individual to perform compensatory motor adjustments for deglutition, as well as the premature escape and the persistence of food residues in the oral cavity. The lack of posterior teeth also causes the reduction in the vertical dimension of the oral cavity, very common in individuals with TMD, and these factors are considered predisposing for the swallowing difficulties in these individuals.

Five individuals (50%) had alterations as to the number of deglutition efforts for different food consistencies, higher than four swallowing efforts. Only 10% underwent eight deglutition efforts to efficiently eliminate the solid food residues from the oral cavity, and 10% required five movements (Chart 1). These findings may result from the limitation of tongue ejection movements guiding the food bolus towards the pharyngeal phase of deglutition. It is very likely that the orofacial pain causes the individual to carry out compensatory motor adjustments in order to perform the task, just like the lack of posterior teeth and the reduction in intraoral proprioception mechanisms corroborate to identify these findings.

Neck and orofacial movements, such as tongue movements against the deglutition of solid and liquid foodstuff, are described in the literature as alterations present in patients with severe TMD^10^, as well as the participation of orofacial muscles, associated neck movements and/or choking movements during swallowing.

The lack of posterior teeth favors the presence of compensatory tongue movements, causing the individual to grind food with the central and lateral upper and lower incisive teeth, as well as utilizing the tongue to perform the compensatory movement against the hard palate, aiding in the masticatory task. In the sample we found that 30% of the individuals did not have this type of alteration and that 20% had a normal swallowing pattern. Moreover, the reduction in mouth opening amplitude may limit even further the masticatory function and deglutition in the oral phase. TMD patients may have pain-related reduced masticatory function.

As to the pharyngeal phase of swallowing, the persistence of food residue in the valleculae was the most frequently observed alteration for all food volumes and consistencies (Chart 2). This finding may also be associated with the lack of dental elements, which would reduce intraoral proprioception, enabling premature food escape to the pharynx, reducing tongue muscle tone, with consequent reduction in the force of ejection and propulsion of the food bolus, peaking in lack of coordination concerning the swallowing process. Moreover, neck and/or orofacial pain seems to be one relevant factor in the development of compensatory motor adjustments both in food compacting as well as in masticatory and swallowing.

The persistence of food residues in the pharyngeal recesses was observed in 40%, and of these, 10% had laryngeal micro-penetration and tracheal micro-aspiration upon the deglutition of liquid, only for the 20 ml volume, being classified as dysphagia (Chart 2). These findings can be justified by anatomical and functional alterations which would favor compensatory motor adjustments and the reduction in intraoral proprioception associated with severe TMD.

In the present study, all the individuals had normal excursion of the laryngeal and hyoid bone anteriorization and elevation movements.

A broader perspective must be considered in multiprofissional investigations and in TMD propaedeutic, considering the importance of the clinical assessment in regards to complaints associated with choking and coughing during meals and reports of diet changes, besides the nutritional conditions of these patients, reflecting a marked weight loss. The clinical history must also investigate episodes of pneumonia, which may be associated with tracheal aspiration^14^.

This study enabled a qualitative analysis of deglutition by means of an exam which does not require additional resources. Videofluoroscopy is an effective propaedeutic resource, since it enables the observation of dynamics images at a reduced speed and without the excessive exposure of the patient to radiation, making it possible to analyze movements in a frame-by-frame basis and the detailed observation of the three phases of deglutition (oral, pharyngeal and esophageal)^15^, ^16^. A careful postoperative follow up of these patients can provide information regarding the results from the procedure, besides valuable information in order to understand the TMD-related dysphagia.

## CONCLUSIONS

Swallowing videofluoroscopy in the oral and pharyngeal phases of deglutition showed the presence of important clinical signs of dysphagia in individuals diagnosed with temporomandibular dysfunction with indication for surgical correction.

This paper suggests the importance of a detailed and careful clinical assessment by the multiprofissional team in caring for these individuals with TMD, gearing attention towards conditions of associated dysphagia, as well as considering the videofluoroscopy assessment as a routine exam and as a means to obtain a more complete diagnosis.
